# Optimization and Multimachine Learning Algorithms to Predict Nanometal Surface Area Transfer Parameters for Gold and Silver Nanoparticles

**DOI:** 10.3390/nano14211741

**Published:** 2024-10-30

**Authors:** Steven M. E. Demers, Christopher Sobecki, Larry Deschaine

**Affiliations:** Savannah River National Laboratory, Aiken, SC 29808, USA; christopher.sobecki@srnl.doe.gov (C.S.); larry.deschaine@srnl.doe.gov (L.D.)

**Keywords:** FRET, NSET, nanoparticles, quenching, optimization, exhaustive grid search, machine learning, multilayer perceptron, Lasso method

## Abstract

Interactions between gold metallic nanoparticles and molecular dyes have been well described by the nanometal surface energy transfer (NSET) mechanism. However, the expansion and testing of this model for nanoparticles of different metal composition is needed to develop a greater variety of nanosensors for medical and commercial applications. In this study, the NSET formula was slightly modified in the size-dependent dampening constant and skin depth terms to allow for modeling of different metals as well as testing the quenching effects created by variously sized gold, silver, copper, and platinum nanoparticles. Overall, the metal nanoparticles followed more closely the NSET prediction than for Förster resonance energy transfer, though scattering effects began to occur at 20 nm in the nanoparticle diameter. To further improve the NSET theoretical equation, an attempt was made to set a best-fit line of the NSET theoretical equation curve onto the Au and Ag data points. An exhaustive grid search optimizer was applied in the ranges for two variables, 0.1≤C≤2.0 and 0≤α≤4, representing the metal dampening constant and the orientation of donor to the metal surface, respectively. Three different grid searches, starting from coarse (entire range) to finer (narrower range), resulted in more than one million total calculations with values C=2.0 and α=0.0736. The results improved the calculation, but further analysis needed to be conducted in order to find any additional missing physics. With that motivation, two artificial intelligence/machine learning (AI/ML) algorithms, multilayer perception and least absolute shrinkage and selection operator regression, gave a correlation coefficient, $R^{2}$, greater than 0.97, indicating that the small dataset was not overfitting and was method-independent. This analysis indicates that an investigation is warranted to focus on deeper physics informed machine learning for the NSET equations.

## 1. Introduction

Förster resonance energy transfer (FRET) is a widely known optical probe method for applications such as drug targeting [1,2,3,4,5], structural biology [6,7,8], biological imaging [9,10,11,12], and energy-transfer studies [13,14,15,16,17,18]. However, the use of metallic nanoparticles (NPs) as fluorescence quenching molecules for FRET arrangements has been shown to have a larger range of energy transfer than standard FRET [19,20,21,22,23,24,25,26,27]. Nanometal surface energy transfer (NSET) has a much larger range of energy transfer than conventional FRET: tens of nanometers compared to tens of angstroms [14,19,21,24,26]. The classical FRET model for energy transfer is founded on nonradiative dipole–dipole interactions between two dyes [26]. Two molecules, a donor and an acceptor, are brought into close proximity and a dipole–dipole interaction occurs to quench the donor emission while enhancing the acceptor’s emission [28]. In surface energy transfer, which NSET is a part of, surface plasmons are the collective oscillations of free electrons within a metal that propagate along its surface [29]. In the case of metallic NPs, these surface plasmons are constrained in the limited volume of the NP and become the localized surface plasmon modes corresponding to the collective oscillations of the whole electron cloud of the metal NP. An image dipole is exhibited by metal surfaces when an electric dipole is in proximity to the surface. This imaging effect is also exhibited in metal NP surfaces [29,30]. This allows the collective, oscillating electrons in the metal NP to form an array of coupled dipoles to the incident dipolar electromagnetic field of the emitting donor molecule that is in close proximity. This coupling of dipole–dipole interactions is analogous to the FRET model but can occur in a range much greater than that of classical FRET [20,21,24,26,28].

In nanoscale application, gold has been used extensively due to its highly tunable plasmonic properties, wide variety of possible geometries, and its resistance to oxidation and other diminishing effects that plague other metal NPs and surfaces. The apt golden standard for plasmonic metal nanostructures is studied in great detail both theoretically [31,32,33,34,35,36,37,38] but also for abundant plasmonic sensing applications across a large number of disciplines [10,20,21,22,23,24,25,26,33,39,40]. Silver nanoparticles (AgNPs), though having to deal with surface oxidation, also have a large range of utility in sensing, plasmonics, and commercial applications [27,32,41,42,43]. These metals are of particular interest for numerous applications, but in this work, pure metal NPs will be examined to test the accuracy of the NSET model using these metals and for their use in NSET applications.

**Figure 1:**
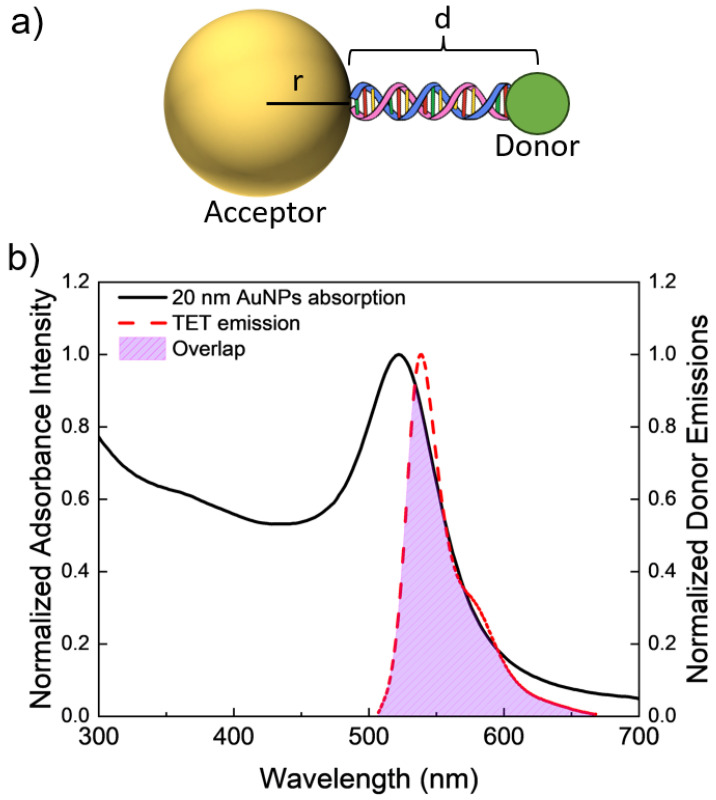
(**a**) Schematic model (not to scale) of a AuNP–DNA–TET dye molecule construct. (**b**) Spectral overlap between the experimentally found acceptor’s (20 nm AuNPs) adsorption spectrum and the donor’s emission. Overlap area is highlighted between the two spectra.

For these metals, a size-dependent NSET model was applied in order to validate the model against the found resonance energy transfer values. The model is tested for a variety of different NPs sizes: for gold nanoparticles (AuNPs) and AgNPs diameters of 10 and 20 nm. Calculations were also performed for AuNPs and AgNPs of 40 nm, copper NPs of diameter 4 and 8 nm, and platinum NPs of diameters 5 and 30 nm, though these were not validated experimentally. These sizes were chosen based on solutions that are sold commercially. To test the effect of distance on the fluorescence quenching efficiency, duplexes of DNA oligonucleotides were attached to the metal NPs’ surfaces that were modified to have an emission donor, the dye tetrachlorofluorescein (TET, $\lambda_{emis}=539$ nm), a fixed distance away from the metal surface. The overlap between the TET dye and a computed 10 nm AuNP solution is presented in Figure 1. Separation is controlled by the number of DNA base pairs within the duplex strands with specific oligochemical modifications allowing for metal surface binding and coupling to the donor.

The focus of this investigation was to examine the NSET model across different metals to allow for a wider range of nanosensor components. The NSET model proposed by Breshike et al. is robust in its handling of different AuNPs that range in size between 0.5 and 20 nm in diameter in the original studies by the group [19,20,21]. However, to allow for a more universal model across different metals, slight modifications were made, stemming from findings of more recent papers that give similar results. It should be noted that the modifications to the Drude free electron model for the dielectric constants of the metals were uniquely represented. Interband transitions occurring in the metals cause the deviations from the Drude model and, as such, must be represented accurately on a case-by-case basis for the metals in the energy transfer calculations. Dye–dye energy transfer interactions were also due to the potential close proximity of adjacent dye–DNA complex strands on the NP surface.

Although biosensor models and experiments are quick and effective tools, there are difficulties in establishing the fluorescence quenching of the sensor materials since there are different coefficients associated with the quenching observed from experimentation. For this reason, various studies have applied artificial intelligence/machine learning (AI/ML) techniques such as multilayer perception (MLP) methods that implement inputs, outputs, and nodes, as well as the least absolute shrinkage and selection operator (Lasso) method to prevent overfitting and reduce errors. On one hand, for the MLP method, neural networking tools were applied towards biosensor application [44,45,46], NP sensors sizes and applications [47,48,49], and FRET sensors [50,51]. Conversely, the Lasso method was applied to prevent overfitting for FRET sensor applications [52,53]. To our knowledge, the NSET model and experimental information have not yet been optimized or undergone AI/ML methods to investigate if there is a high correlation between the inputs’ attributes and fluorescence quenching.

Two variables, *C* (from the size-dependent damping constant term) and α (the orientation of the donor to the metal plasmon vector), were varied via the exhaustive grid search (EGS) optimization algorithm to determine the optimal values followed by AI/ML modeling using MLP and Lasso methods. For both algorithms, 56 cases were investigated that focused on Ag and Au NPs and the parameters involved in the NSET model and fluorescence quenching. For each ML algorithm, three studies were conducted to calculate the correlation coefficient ($R^{2}$), prove that overfitting was prevented, and confirm that the dataset was method-independent. The article then concludes that additional investigations need to be conducted for a more complete study to improve upon the nanosensor applications and the physics of the proposed modeling.

## 2. Materials and Methods

### 2.1. Clegg Model DNA Distances

Modified oligonucleotides were obtained from Integrated DNA Technologies Inc. (Coralville, IA, USA) as lyophilized powders and resuspended in phosphate buffer. Double-stranded DNA (ds-DNA) of different lengths were utilized to control the spacing between donors and metallic NPs by assuming a rigid rod behavior of the ds-DNA. The DNA lengths used in the experiments are 20, 40, 60, and 80 base pairs (bp). For the NP-connecting strands, a PEG3 spacer connects the DNA strands to a thiol 5′ end. For the donor DNA strands, the same spacer connects the conjugate DNA strands to the 5′ TET dye molecule. These lengths correspond to NP surface to center of the donor distances of 95 Å (20 bp), 163 Å (40 bp), 231 Å (60 bp), and 299 Å (80 bp), as calculated by the Clegg model and taking into account the spacers and binding terminals [54]. See Supplementary Materials Sections S1 and S4 for the sequences used in the DNA strands and calculations for the oligo-modification lengths.

### 2.2. DNA Conjugation

Citrate-capped 10, 20, and 40 nm gold, 10, 20, and 40 nm silver, and 5 and 30 nm platinum NPs were purchased from Sigma-Aldrich (St. Louis, MO, USA) and used as is. Copper NPs can also be purchased from Millipore-Sigma, but these colloid solutions require purification and citrate encapsulation for use in experiments. Instead, it is suggested that one follow the synthesis method of Yu et al. [55] to produce monodisperse copper nanoparticles that can undergo ligand exchange to citrate. Sodium chloride (NaCl), tris(2-carboxyethyl)phosphine (TCEP), monobasic and dibasic sodium phosphate, citric acid, and sodium citrate were purchased from Sigma-Aldrich (St. Louis, MO, USA) and used without further purification. Phosphate buffer (PB) was created from monobasic and dibasic sodium phosphate. Phosphate buffer saline (PBS) was made by dissolving 0.1 M NaCl in 0.01 M PB. Citrate buffer (0.25 M, pH = 3) was made from citric acid and sodium citrate.

The lyophilized, modified oligonucleotides were first resuspended in PB and then brought to a concentration of 0.1 M TCEP. The DNA solutions were allowed to incubate for 1 h. The solutions were then purified with an Illustra Nap 5 column (GE HealthCare, Chicago, IL, USA) and the end suspension was PB. The solutions were then stored at −30 °C until needed for NP conjugation.

For gold NP–DNA conjugation, the procedure described by Hurst et al., was followed [56]. Briefly, roughly 1.5 mL of the (~1 OD) stock AuNP solution was concentrated to 100 μL using centrifugation. The concentration was determined using absorbance measurements [57] and then between 100, 300, or 600 molar excess activated thiol–DNA strands were added to the concentrated solutions and the final solution was adjusted to 0.01 M PB. An 11-step slow salt aging process then occurred to a final NaCl concentration of 1 M. After each NaCl addition, the solutions were sonicated for 10 s and then shaken for 20 min to facilitate the thiol–Au binding. The solutions were left to incubate overnight. To remove excess DNA, the conjugated AuNPs were purified using a 50 kDa Amicon spin filter (Sigma-Aldrich, St. Louis, MO, USA) and resuspended in PBS. The filtrate was removed, and the concentration of the unbound DNA in the filtrate was compared to control complementary strands without AuNPs using UV–Vis.

For the 10 nm AgNP–DNA conjugation, a citric acid technique was used instead [58]. A similar 100 μL concentrated solution was obtained and 200 molar excess thiol-activated DNA was added to the solutions. The solutions were then brought to 5 mM citrate buffer. The solutions were sonicated for 10 s and then shaken for 20 min. The solutions were then brought to 10 mM citrate buffer and shaken for 25 min. They were then spin column purified.

### 2.3. Energy-Transfer Measurements

Fluorescence and UV–Vis measurements were collected using a Horiba Duetta spectrometer (Kyoto, Japan). UV–Vis measurements were attained with 5 nm bandpass and 0.05 s integration. Fluorescence measurements used an excitation of λ=522 nm with a collection wavelength range of 525–700 nm, 10 accumulations, and 10 s integration per sample.

The NP–DNA–TET solutions were created by mixing the QDs–DNA solution to the conjugate DNA–TET solution in a ratio of approximately 50:1, 90:1, and 180:1 for the 10, 20, and 40 nm NPs, respectively. These ratios were chosen because they are roughly two-thirds of the amount of DNA that is bound on the surface of these NPs when they are fully saturated with thiolated DNA [59]. An equal amount of just DNA–TET solution for each of those conjugates with equal amounts of nonconjugated metal NPs was set aside as well for a baseline emission measurement. The solutions’ mixtures were then shaken for 1 h to allow for full coupling of the conjugate DNA strands to occur. The NP–DNA–TET solutions were not purified to allow an equal comparison to the baseline measurement.

It has been noted in the previous literature such as Chen et al. [60] for AuNPs that fluorescent molecules near a AuNP surface exhibit an NSET-like response even with increasing amount of fluorophores near the NP surface. In quantum-dot-based complexes, the close proximity of adjacent dyes allow for nonquenching-based decay pathways other than the desired QD–dye FRET quenching [61,62]. However, it would be prudent to note that the duplexed DNA strands connected to the NP surface have some limited mobility to bring separate dye molecules to close proximity to one another. These alternative decay pathways will be discussed in greater detail later.

## 3. Energy Transfer Equations

### 3.1. FRET Equation

The Förster distance ($R_{0}$) is the distance when 50% of the nonradiative quenching of the donor fluorescent molecule is reached. This Förster distance be expressed in many different units, but for the purpose of this work and the Python script, the equation from Lakowicz is used [28]:

(1) $$R_{0}=0.211{\left(\kappa^{2}n^{-4}Q_{D}J\left(\lambda \right)\right)}^{1/6}$$

where $\kappa^{2}$ is relative orientation factor in space between the donor and acceptor dipoles (here assumed to be 2/3), *n* is the refractive index of the medium, $Q_{D}$ is the quantum yield of the donor in the absence of acceptor, and the overlap integral J(λ) is given by

(2) $$J\left(\lambda \right)=\int_{0}^{\infty }F_{D}\left(\lambda \right)\epsilon_{A}\left(\lambda \right)\lambda^{4}d\lambda$$

with $F_{D}\left(\lambda \right)$ being the corrected fluorescence intensity of the donor in the range of λ+dλ with total intensity normalized to unity, and $\epsilon_{A}\left(\lambda \right)$ is the extinction coefficient of the acceptor at each λ in units of M^−1^ cm^−1^. For the Python script, the dλ values were set to 1 nm increments for the purposes of calculations. While the $\kappa^{2}$ value could have been varied, it was not during AI/ML optimization to keep a constant comparison for the NSET variations.

### 3.2. NSET Formula

The NSET equations proposed by Breshike et al. are used when an emitting fluorescent molecule or particle are quenched by a metallic nanosphere. These equations are a modification of CPS–Kuhn theory [20,21,63], whereby a dipole has a rate of nonradiative energy transfer to the metal NPs’ conduction electrons in a surface energy transfer [64]. Yun et al. also point out that there are three different different interactions regimes between the fluorophore molecules and the metal NP surface: (1) at less than 10 Å there is radiative rate enhancement, (2) between 20 and 300 Å energy transfer dominates, and (3) at distances > 500 Å dipole–mirror effects cause fluorescence oscillations [64]. For the purpose of this work and whose calculations are in Supplementary Section S4, the distances between the fluorescent dye and that of the metal NP are in the intermediate domain and range from 76 to 330 Å and, thus, the nonradiative energy transfer of NSET dominates. Of practical relevance, the NSET model has a distance dependence of $1/r^{4}$ instead of the $1/r^{6}$ that is usual for FRET calculations. The type of nonradiative transfer between donor fluorophore and acceptor NP in DNA constructs determines the fluorescent quenching slope observed in experiments.

For NSET interactions, the distance dependence is best described in terms of the distance ($d_{0}$) at which the probability of nonradiative quenching of the fluorescent molecules excited state results in a lowering of the PL intensity by 50%:

(3) $$d_{0}=\frac{\alpha \lambda_{emis}}{n_{p}}{\left(A\Phi \right)}^{1/4}{\left(\frac{n_{m}}{2n_{p}}\left(1+\frac{\epsilon_{1}^{2}}{|\epsilon_{2}{|}^{2}}\right)\right)}^{1/4}$$

where α is the averaged vector of the donor to the metal plasmon vector resulting in $\alpha =({\left(9/2\right)}^{1/4})/4\pi$, $\lambda_{emis}$ is the emission wavelength maximum of the donor, $n_{p}$ is the index of refraction solvent (double-stranded DNA, $n_{p}=2.5046$) [65], Φ is the quantum yield of the donor, $n_{m}$ is the index of refraction of the metal at the donor emission wavelength, $\epsilon_{1}$ is the solvent dielectric ($\epsilon_{1}=\epsilon_{DNA}=1.5881$) [65], and $\epsilon_{2}$ is the complex dielectric function of the metal that can be decomposed into real and imaginary parts ($\epsilon_{2}=\epsilon_{2^{\prime }}+i\epsilon_{2^{\prime \prime }}$). The modification from the CPS–Kuhn expression accounts for the alterations to the mirror-like behavior of gold as it is reduced in size to nanometers. Breshike et al. accounted for the size-dependence in the equations they presented, but publications since this paper have given expressions that allow for a wider range of metals to be examined [20].

The NP absorptivity (*A*) and metal complex dielectric function ($\epsilon_{2}$) become size-dependent in the regime of <50 nm for metal NPs, and one must account for this size dependence. From Breshike et al. [20] the absorptivity of a single NP can be formulated as

(4) $$A_{np}=10^{3}ln\left(10\right)\left(\frac{\epsilon_{\lambda }\left(2r_{cm}\left(\frac{2r_{cm}}{\delta_{skin}}\right)\right)}{N_{A}V_{cm^{3}}}\right)$$

where $\epsilon_{\lambda }$ is the extinction coefficient of the NP at the maximum emission wavelength of the donor, $r_{cm}$ is the radius of the NP in cm, $N_{A}$ is Avogadro’s number, $V_{cm^{3}}$ is the volume of the particle in cm, and $\delta_{skin}$ is the skin depth, discussed later. The extinction coefficient $\epsilon_{\lambda }$ is calculated directly from the Mie theory for an NP of a particular size and composition [33].

### 3.3. Modification of NSET Formula for Additional Metals

The complex dielectric function of the metal ($\epsilon_{2}$) comprises the dielectric terms for the bulk ($\epsilon_{\infty }$), Drude ($\epsilon_{Drude}$), and the interband transition ($\epsilon_{IB}$) contributions for each metal discussed in this report, that is, $\epsilon_{2}=\epsilon_{\infty }+\epsilon_{Drude}+\epsilon_{IB}$. The Drude term, $\epsilon_{Drude}$, can be expressed as the following:

(5) $$\epsilon_{Drude}=\epsilon_{\infty }-\frac{\omega_{p}^{2}}{\omega^{2}+\Gamma_{r}^{2}}+i\frac{\Gamma_{r}\omega_{p}^{2}}{\omega (\omega^{2}+\Gamma_{r}^{2})}$$

where ω is the dipole frequency, $\omega_{p}$ is the Drude plasmon frequency of the metal, and $\Gamma_{r}$ is the size-dependent damping constant. Breshike et al.’s formulation of the size-dependent dampening constant was ($\Gamma_{r}=\Gamma_{\infty }+\left(l_{\infty }\right)/r$) where ($l_{\infty }=420$ Å) for AuNPs of radius, *r*. To allow for computational determination of NPs of other metals, the size-dependent damping constant was changed to one found in Bohren and Huffman [66,67]

(6) $$\Gamma_{r}=\Gamma_{\infty }+C\frac{v_{f}}{r}$$

where $\Gamma_{\infty }$ is the bulk dampening constant of the metal, *C* is the scattering constant that details the scattering process (values range from 0.1 to 2; initially 0.33 was used), $v_{f}$ is the electron velocity at the Fermi surface of the metal, and *r* is the radius of the NP. C=0.33 was the approximate value similar to Breshike et al. [20] and was used as its initial value.

Next, the penetration or skin depth of the oscillation dipole of the NP is the distance at which electromagnetic radiation will penetrate into a metal [29]. A simple relation for the skin depth for a metal to first order by Liu and McLeod [68] was used in this study to allow for a uniform calculation of this property across different NP compositions. The relation is given by

(7) $$\delta_{skin}=\lambda /\left(2\pi \epsilon_{2^{\prime \prime }}\left(\lambda \right)\right)$$

where $\epsilon_{2^{\prime \prime }}$ is the imaginary part of the refractive index for the material, assuming unit relative magnetic permeability. While this expression is not size-dependent, as seen in Breshike et al.’s results [20], this expression gives similar skin depths that are applicable to the different metallic NPs that are presented in this work.

Finally, there was a modification to the dielectric terms to better account for the interband transitions of NPs. As stated in numerous other findings, interband transitions make significant contributions in the 300–500 nm wavelength region and cause the discrepancy from the Drude model in this region for the gold dielectric [34,69].

To account for this difference, Breshike et al. [20] introduced the following interband contribution to the complex dielectric function [20]:

(8) $$\epsilon_{IB}=\sum_{i=1,2}\frac{A_{i}}{\omega_{i}}\left[\frac{e^{i\Phi_{i}}}{\omega_{i}^{-1}-\omega^{-1}-\Gamma_{i}^{-1}}+\frac{e^{-i\Phi_{i}}}{\omega_{i}^{-1}+\omega^{-1}+\Gamma_{i}^{-1}}\right]$$

where $A_{i}$, $\omega_{i}$, $\Phi_{i}$, and $\gamma_{i}$ are fit parameters in frequency given by the authors.

Another proposal by Derkachova et al. uses a logistic function to append to the Drude imaginary term ($\epsilon_{2}\left(\omega \right)=\epsilon_{Drude}\left(\omega \right)+i\Delta \epsilon^{\left(Au\right)}\left(\omega \right)$) with [36]:

(9) $$\Delta \epsilon^{\left(Au\right)}\left(\omega \right)=\frac{A}{1+exp(\left(\omega_{c}-\omega \right)/\Delta )}$$

where A=5.6 eV and δ=0.17 eV are the fit parameters to the Johnson and Christy experimental dataset [34]. This logistic function is accurate for the region of 1–3 eV, which allows for accurate representation of the dielectric function at the emission wavelength of the TET dye at 539 nm (2.3 eV). The other necessary terms for the gold dielectric are taken from Oubre and Nordlander [70], with $\epsilon_{0}=9.5$ eV, $\gamma_{\infty }=0.06909$ eV, and $\omega_{p}=8.9488$ eV. The wavelengths for the emission of the donor molecules were converted to eVs in the Python calculations to keep units consistent for the computation. For a comparison of the different dielectric models, please refer to Supplementary Section S2.

It becomes apparent that consideration is needed to account for the interband transitions within the AuNPs as a function of the NP size. A general equation is needed that accounts for nanoscale size effects uniformly across the different metal NPs. Here, a size-dependent dielectric multiplication factor was created by comparing the size-dependent Drude components to the bulk metal dielectric function:

(10) $$\epsilon_{i,Multiplier}=\frac{\epsilon_{i,radius}}{\epsilon_{i,\infty }}=\frac{\epsilon_{\infty }-\frac{\omega_{p}^{2}}{\omega^{2}+\Gamma_{r}^{2}}}{\epsilon_{\infty }-\frac{\omega_{p}^{2}}{\omega^{2}+\Gamma_{\infty }^{2}}},\frac{i\frac{\Gamma_{r}\omega_{p}^{2}}{\omega (\omega^{2}+\Gamma_{r}^{2})}}{i\frac{\Gamma_{\infty }\omega_{p}^{2}}{\omega (\omega^{2}+\Gamma_{\infty }^{2})}}$$

where the size dependent term $\gamma_{r}$ uses the modified, sized-dependent dampening constant of Equation (4), and $\gamma_{\infty }$ is the bulk term for the same metal. There are two multiplication factors, since $\epsilon_{i}=\epsilon_{{i,}^{\prime }}+i\epsilon_{{i,}^{\prime \prime }}$, and so each dielectric term must be calculated independently of one another, though they follow the same form. These modifications to the dielectric terms account for the interband transitions normally neglected in the Drude model while still retaining the NP size dependence. A comparison between the models can be seen in Supplementary Section S3.

## 4. Results

### 4.1. Modified NSET Model Experimental Studies

The extinction spectrum and the resulting NSET/FRET distances were calculated by determining the values for Equation (9) at each wavelength and multiplying the found $\epsilon_{i,Multiplier}$ by the experimentally found dielectric value from Johnson and Christy [34]. The Mie scattering solutions for metal NPs were calculated from the modified Johnson and Christy dielectric values and the resulting absorbance cross-sections were determined. The expected NSET $d_{0}$ values were then calculated by plugging the found dielectric values at 539 nm, Equations (4) and (5) into NSET Equations (1) and (2). The expected FRET calculations for the same TET dye donor and metal NP acceptor were then calculated using the standard FRET equation [28]. All of the values determined from the calculations can be found in Table 1 for a TET dye donor emitting at 539 nm. The values chosen for the NP sizes are those that can easily be bought through commercial sellers of NP products.

**Figure 2:**
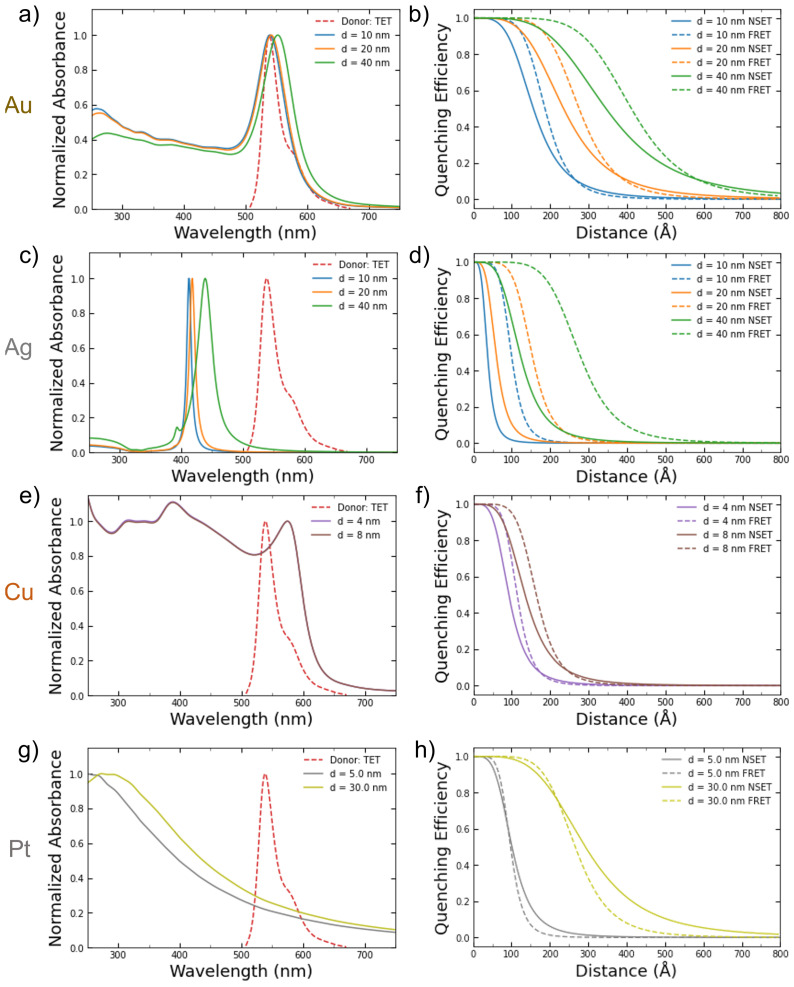
Calculated absorbance spectrums for (**a**) gold, (**c**) silver, (**e**) copper, and (**g**) platinum NPs of varying commercially available or easily synthesized sizes compared to the emission spectrum of the TET dye. The resulting calculated FRET and NSET fluorescence quenching efficiencies for those same-sized (**b**) gold, (**d**) silver, (**f**) copper, and (**h**) platinum NPs.

Figure 2a,c,e depict the calculated absorbance spectrum of the NPs for the various sizes with the emission peak of the TET dye donor overlaid. TET was chosen since it has a large overlap with the gold and copper NPs in the sizes examined in this work. Since the silver NPs’ absorbance peaks (~420 nm) are so far away from the TET emission peak at 539 nm, they serve as a good baseline for undesirable effects such as aggregation that might occur during the DNA conjugation process. The calculated NSET and FRET distance vs. fluorescence quenching efficiency curves for the same metal NPs sizes are shown in Figure 2b,d,f.

### 4.2. Gold Experimental Results

The 10 nm AuNPs had the varying lengths of DNA conjugated to their surface following the procedure mentioned before. To account for possible scattering effects, the absorbance and emission of the same concentration of unmodified 10 nm AuNPs was taken. The concentration of each of the AuNP–DNA–TET solutions was calculated based on the absorbance peak of gold’s plasmon resonance at 525 nm. The concentration was compared to the unmodified gold and a relative concentration factor was found. This factor was multiplied to the emission spectrum at all wavelengths to obtain an emissions background spectrum. The emission background spectrum was then subtracted from the AuNP–DNA–TET emission spectrum, allowing for the determination of just the TET emissions for each sample. This same background process was applied to every NP solution’s measurement. Refer to Supplementary Section S5 for additional information.

**Figure 3:**
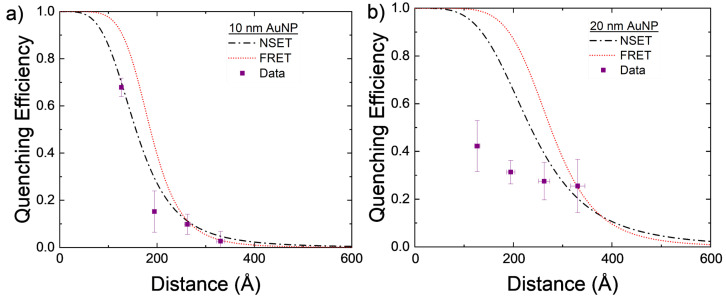
Calculated NSET and FRET values for AuNPs of size (**a**) d = 10 nm and (**b**) d = 20 nm, compared to experimentally found values. Each DNA length was measured four times with the mean and standard error displayed. The distance error bars are partially covered by the symbols.

The experimentally found values for AuNP–DNA–TET are compared to the calculated NSET and FRET values in Figure 3a. While both the calculated NSET and FRET fluorescence quenching efficiencies are close to one another, the experimental data appear to more closely follow that of the NSET prediction.

The 20 nm AuNPs were also investigated using the same DNA–TET strands as for the 10 nm AuNPs. With the 20 nm particle, the scattering from the excitation was significant but by using the background correction described previously, the TET emission can be elucidated. The data gathered from these experiments do not follow either the NSET or FRET models for 20 nm AuNPs. Several things may account for this discrepancy, for example, the DNA strands not being at the same effective length as previously believed [59,71], full hybridization of DNA was not achieved due to the tilting of the DNA on the gold’s surface [59], or a plasmon resonant energy transfer (PRET) effect may be beginning at these NP sizes. As mentioned in Section 2, dye–dye interactions may also allow for a nonenergy transfer decay path to be created in adjacent dye molecules that are within close proximity to one another. In the larger NPs, DNA strands may be at a more tilted orientation and, thus, allow for more dye–dye interactions to occur. These nonenergy transfer decay paths may lower the observed quenching observed in the complete NP–DNA–dye system.

The 40 nm AuNPs were attempted as the upper bound for NP size due to the prevalence of PRET at larger NPs sizes. However, a tremendous amount of scattering was observed using the same 539 nm excitation as the other two AuNP sizes. This scattering completely overtook any measurable response for the TET dye emission, and a background subtraction was not feasible. If the 40 nm AuNPs were to be tried again in the future, then a different donor molecule would need to be chosen that has an absorbance that is not strong as that of the plasmon resonance of the NPs.

### 4.3. AgNP Findings

While AgNPs are used in a variety of different commercial and medical applications, a thorough understanding of the changes to the dielectric function as a function of AgNPs across a wide range of wavelengths has yet to be compiled. Interband transitions below 350 nm contribute to the plasmonic capabilities of the NPs, which, similar to gold, causes discrepancies from the Drude free-electron models. To account for the interband transitions, Equation (9) was applied to the bulk silver dielectric function found by Yang et al. [41]. The modified bulk dielectric function values were then used in the original FRET equation in order to determine the Förster distance in comparison to the calculated NSET values.

**Figure 4:**
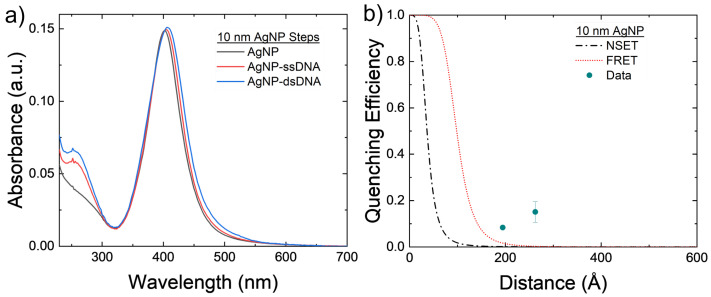
(**a**) Absorbance spectrum of the 10 nm AgNPs at varying steps of the conjugation process for the 60 bp DNA strands. Notice the small red shifting of the main plasmon peak, possibly coming from AgNP aggregation. (**b**) Comparison of experimental 10 nm AgNP–TET to FRET and NSET models. Averages and standard errors from 3 measurements are plotted.

The 10 nm AgNPs were conjugated with the 40 and 60 bp DNA strands to test both NSET and FRET models for nonoverlapping emission and donors. The results can be seen in Figure 4. Figure 4b shows that there is minimum fluorescence quenching observed in the system caused by either FRET or NSET. The roughly 10% that is seen in both arrangements may be caused by a slight amount of aggregation of the AgNPs that can be seen in Figure 4a. These larger silver clusters act as effectively larger AgNPs, which causes both the NSET and FRET distances to increase and, thus, increase the quenching observed in the NP–TET acceptor–donor system, as seen in Figure 2d.

Overall, the NPs across the different metals and sizes tested more closely follow the fluorescence quenching predicted by nanometal surface energy transfer equations than for Förster resonance energy transfer. Overall, NSET appears to be the dominating interaction between donor molecules and NPs with sizes between 2 and 20 nm in diameter [20]. With the framework assembled here, this same analysis may be applied to metal NPs of catalytic importance (Cu, Ni, Pd, Pt) as well as different metal alloys and combinations material shells. Additionally, a more thorough examination into the electronic properties of quantum dots may also be addressed in the future.

## 5. Exhaustive Grid Search Optimization

The optimization tool applied in this study was the EGS method for both AgNPs and AuNPs. The EGS uses a full-factorial sampling “grid” over one or more parameters, in which the “exhaustive search” is applied. For each parameter, a sequence is also supplied as a space-separated list of dimensional values. The EGS method in this study was applied because the Python script ran quickly enough on a desktop/laptop where thousands of different parameter combinations can run and a defined “grid” can be visualized. This method was selected because EGS is effective for smaller datasets and fewer parameters, and, at the same time, promotes automation by minimizing intervention in tuning the NSET model. In this study, for 56 simulations, the EGS method optimized the *C* and α values with different dimensions.

The criteria to measure the network performance relied on the $R^{2}$, mean relative errors (MRE), and the root mean square error (RMSE). The measured outcome provided the verification of the assumptions and lists made. The coefficients were first set to a range and ran in a loop and calculated the power output for each of the 56 simulations (52 Au and 4 Ag particles). The EGS method continued until all combinations of α and *C* values were completed. The MRE estimates the percentages between the error and the experimental values:

(11) $$MRE\left(\%\right)=\frac{1}{N}\sum_{i=1}^{N}\left|100\frac{Q_{c}-Q_{a}}{Q_{a}}\right|,$$

where *N* is the number of points in the given dataset, and $Q_{c}$ and $Q_{a}$ are fluorescence quenching based on the computation and measurements, respectively. This MRE was followed by the RMSE:

(12) $$RMSE=\sqrt{\frac{1}{N}\sum_{i=1}^{N}{\left(Q_{c}-Q_{a}\right)}^{2}}.$$

In the current study, a total of 56 calculations ran on the particle features and other parameters that calculated the NSET and FRET fluorescence quenching values. The inputs varied between the metal and size of the NP, orientation angle, DNA length, observed fluorescence, NSET and FRET parameters, and other theoretical values, as seen in Table 2. The only constant values were the size error, *C*, and α values, in which the latter two changed as the main focus of the optimization study. By using the provided measurements and values, the fluorescence quenching output for NSET and FRET were compared to the true fluorescence quenching value.

**Figure 5:**
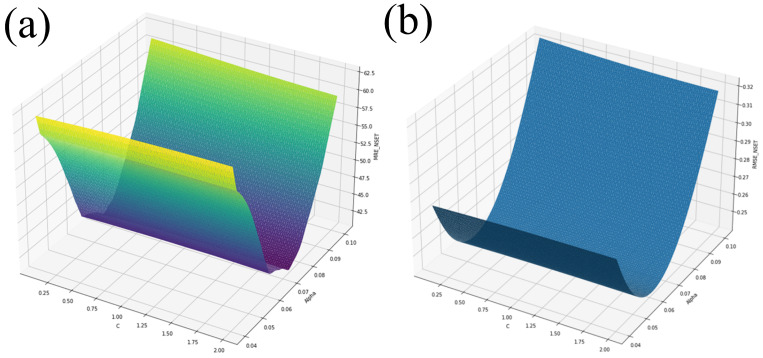
EGS for 0.04≤α≤0.1 and 0.1≤C≤2.0 for (**a**) MRE and (**b**) RMSE.

Three EGS ran, with each set of *C* and α parameters running the Python script containing the NSET equations. The search started from a coarse grid with a wider band of α and *C* values, and became finer with a narrower band for each run. The ranges were 0.0≤α≤4.0 and 0.1≤C≤2.0, 0.04≤α≤2.0 and 0.1≤C≤2.0, and 0.04≤α≤0.1 and 0.1≤C≤2.0. Each time, 56 cases ran quickly, and each EGS was performed until the *C*, α, MRE, and RMSE values improved very little. The three EGS runs resulted in over 1 million calculations, resulting in an efficient performance of a complete enumeration and, hence, showing the complete response function. Figure 5a,b show the results of the EGS method for 0.04≤α≤0.1 and 0.1≤C≤2.0.

The *C* and α results and errors for each grid optimization search can be seen in Table 3. As observed, when moving from the first to second grid searches, the MRE and RMSE values decreases significantly. Afterwards, when moving from the second to third grid searches, the MRE and RMSE values not only barely differed, but the *C* coefficients did not change while α was not tuned significantly.

The initial and final grid search parameters are plotted alongside the base NSET and FRET calculated fluorescence quenching values in Figure 3 and Figure 4 with dashed blue and green lines, respectively. Some improvements were made on an individual and physics modeling basis from the initial NSET parameters used, though perhaps in the future the different metals would need to be considered individually with respect to C and α if additional trials can be conducted for silver, copper, and platinum NPs.

**Figure 6:**
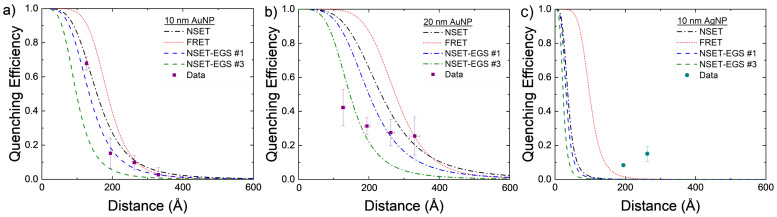
Experimental values compared to calculated FRET (red dots), initial NSET value (black dashes), and final fitted NSET values (dashes are the best optimization results from the exhaustive grid searches) for (**a**) 10 nm AuNPs, (**b**) 20 nm AuNPs, and (**c**) 10 nm AgNPs.

Further analysis can be seen in Figure 6 by comparing each EGS result to the SME parameter values for AuNPs and AgNPs. Some were able to improve on an individual and a physics modeling basis, but the physics must be improved.

## 6. Machine Learning Applications and Results

Two machine learning applications were applied to 56 studies: multilayer perceptron (MLP) and the least absolute shrinkage and selection operator (Lasso) using SciKit Learn version 1.2.2. The two methods were selected as part of a screening step by running many other ML algorithms and comparing $R^{2}$ and their respective errors. The MLP and Lasso methods had the highest $R^{2}$ and least errors, which resulted in their selection and seeing if any further fine-tuning can further improve the analysis.

The MLP method is a popular and well-known ML tool that is similar to human brain functions for computerized training mechanisms [73]. This method was chosen for its ability to solve a wide array of problem types, especially in biology and sensor applications previously mentioned in Section 1. By inputting the observed and computed variables into the analysis, the MLP technique adjusts its weights and iterates to find the best predicted output through a training–retraining process. Results are then validated on unseen (held out) data.

The Lasso method is applied as a method to regularize linear regression models and reduces errors caused by overfitting on the training data [73]. The Lasso method is often used as an ML technique to handle high-dimensional data by adding a penalty term to the residual sum of squares, and it is then multiplied by the regularization parameter, which controls the amount of regularization applied. By increasing the penalty, the Lasso method can reduce the importance of some of the features from the model, which assists in feature selections. In sensor methods, the Lasso regression technique was also applied in FRET applications as mentioned in Section 1.

In the current study, Table 2 lists the variables and ranges of those variables used to calculate the NSET fluorescence quenching. The wide range of values for each variable was standardized between 0 and 1 for the MLP and Lasso algorithms and were then imported to the Scikit Learning program for training and validation. The only change to the dataset is that the NP metals Au and Ag were reclassified as 0 and 1, respectively. To ensure that no overfitting is taking place, three runs calculated the $R^{2}$ results. The first case ran a standard 2/3rd training to 1/3rd testing. The second case ran a 5-fold cross-validation (CV) study to find the hyperparameters that minimize errors or other performance metrics. Here, 10% was removed from the analysis, the model was developed, and then the results on the fully withheld 10% were evaluated.

**Figure 7:**
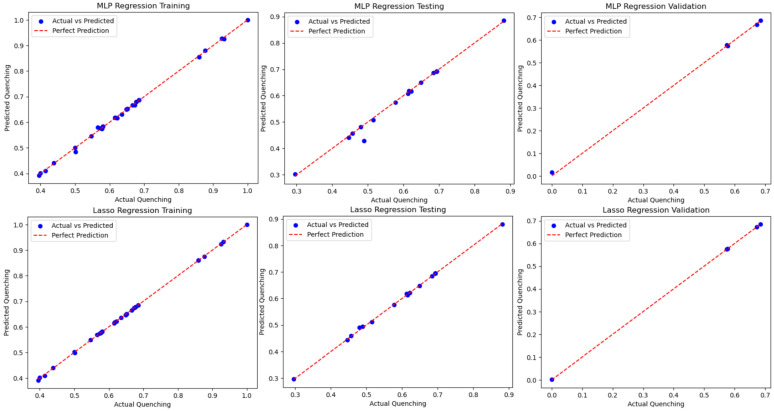
Blind test of the MLP and Lasso models with 10% validation data extracted from the total data.

The results in Table 4 show that for each case, $R^{2}>0.97$ for both methods, indicating that not only is the current dataset method-independent, but the methods applied are not overfitting. Further results can be seen in Figure 7 when comparing the methods and the training, testing, and validation results on a best-fit line. Except for a few outliers in the MLP training set, almost all datasets fit almost perfectly on the best-fit line. The resulting study uncovered a high-fidelity function relationship, and a study including explainable AI would be needed to begin to identify the relationship that the ML algorithms discovered. Additionally, the subject matter expert can assess if these relationships can assist in the physics-based discovery of the NSET model.

## 7. Conclusions

Nanometal surface energy transfer appears to be more accurate than Förster resonance energy transfer for the acceptor–donor interaction of different metal NPs with the fluorescent dye TET. Slight refinements to the original NSET equations allow for the equation to be more broadly applied to different metal NPs other than those of just gold, allowing for a greater possibility of donor–acceptor pairs. NSET seems to predominate for acceptor NPs in the rough range of 2–20 nm diameter when interacting with donor molecules, though significant scattering and other effects start to appear at a diameter of 20 nm. These NP–donor systems can be expanded upon to determine if NSET is, indeed, the primary acceptor–donor fluorescence quenching mechanism for metal NPs. Biosensors can utilize different metal NPs in their design for a greater variety of properties that detect analytes in biological or environmental systems and other potential applications.

Multiple ML techniques, i.e., MLP and Lasso algorithms, contributed in the increased accuracy of fluorescence quenching levels from the current study by producing multiple results where $R^{2}>0.97$. Initially, the *C* and α values were optimized as an improvement to increase the NSET equation’s accuracy by comparing the best-fit curves to the experimental results. After running over 1 million calculations with three exhaustive grid searches, the NSET equations improved significantly for AuNPs, but not for AgNPs. Furthermore, the errors improved, but were relatively high, which motivated further study of ML methods to increase the correlation and help to inform further studies to find the missing physics or optimize each of the metal NPs. Focusing on the MLP and Lasso algorithms, three cases provided similar accuracy by running a standard 2/3rd training 1/3rd testing, a 5-fold cross-validation study, and a blind test study where 10% of the original data were extracted. On one hand, the MLP study was able to apply the weights and attributes to identify that there is a strong correlation between the input attributes and fluorescence quenching. Conversely, the Lasso study prevented overfitting and also showed a very strong correlation. Both algorithms demonstrated the ability to increase the accuracy and, hence, applicability, of this approach for fluorescence quenching by using theoretical and experimental information. One question that remains, however, is if the accuracy could be improved with smaller or larger datasets. In this research, 56 cases were studied using MLP and Lasso algorithms, which gave a concern that overfitting could occur, even though this study proved that this was not the case. Additionally, the results are method-independent since both ML algorithms were successful.

For the physics model, there were cases in which the NSET equations overestimated or underestimated the actual fluorescence quenching. This is attributed to two different factors. (1) The 20 nm gold NPs surface-bound DNA strands are at a more extreme tilt (40°) than the smaller 10 nm NPs at near normal to the NP surface. This could potentially make it much harder for the hybridization of the two DNA strands near the NPs’ surface, due to the electrostatic repulsion of adjacent phosphate backbones. (2) The silver NPs, which were supposed to be at near 0 quenching, show that NP aggregation could be present even with careful surface conjugation. These observed experimental variances as well as others could lead to optimized values of *C* and α that differ from this study’s findings.

To further improve the ML algorithms and the NSET integrated approach, a single metal NP type could be used as an experimentation test bed. For example, it can be used to determine the necessary (i.e., minimum) number of cases to be collected to generate reliable results. If a metal type has a large range of data, how many data points are necessary for the MLP and Lasso studies? These results could also assist with efficient equation development for physics-informed ML, making the physical case more complete and comprehensive. An additional case study using this dataset could be applied in design of experiments to explain the influence between the fluorescence quenching, DNA length, orientation, etc., in order to identify and explain the control variables.

Further action can improve the current work for applications in the NSET models by answering the following questions:

- What did the MLP and Lasso algorithms uncover from these highly accurate results and what was the physics knowledge gained?
- What could more than 56 datasets reveal from the same metal NPs to anticipate the amount of sufficient data, and would different hyperparameter setups for MLP and Lasso be required?
- Can *C* and α values improve based on the weighted contribution on the individual metal NPs as opposed to treating the metal NPs the same?
- What would explainable AI reveal for fluorescence quenching results?
- Would MLP and Lasso algorithms be particle-specific if other metal NPs (e.g., Cu, Pt, Ni, Pd, Al, W) were assessed?

## Figures and Tables

**Table 1:** Metal NPs and their parameter values.

		$\lambda_{\mathrm{ex}}=539$ nm		$\epsilon_{2}=\epsilon_{2}^{\prime }+i\epsilon_{2}^{\prime \prime }$		$d_{0}$ (Å)		
Metal	NP Diam (10^−7^ cm)	NP Extinction (M*cm)^−1^	$\epsilon_{2}^{\prime }$	$\epsilon_{2}^{\prime \prime }$	$|\epsilon_{2}{|}^{2}$	Overlap Int $\frac{{\mathrm{nm}}^{4}}{M*\mathrm{cm}}$	NSET	FRET
Gold	40	$9.46\times 10^{9}$	−5.08	2.63	32.76	$7.32\times 10^{20}$	345.32	411.40
	20	$1.02\times 10^{9}$	−4.98	3.05	34.11	$6.56\times 10^{19}$	234.89	275.89
	10	$1.01\times 10^{8}$	−4.75	3.80	36.95	$6.35\times 10^{18}$	156.41	186.52
Silver	40	$8.76\times 10^{8}$	−12.09	0.55	146.42	$6.51\times 10^{19}$	120.59	274.89
	20	$2.46\times 10^{7}$	−11.93	0.70	142.70	$1.84\times 10^{18}$	58.75	151.67
	10	$1.81\times 10^{6}$	−11.50	0.97	133.23	$1.37\times 10^{18}$	36.57	98.43
Copper	8	$1.99\times 10^{7}$	−4.82	8.76	99.96	$2.93\times 10^{18}$	134.56	163.95
	4	$2.44\times 10^{6}$	−3.91	10.49	125.39	$3.32\times 10^{17}$	92.32	114.07
Platinum	30	$6.12\times 10^{8}$	−8.93	18.05	405.53	$2.37\times 10^{10}$	292.65	266.17
	5	$1.49\times 10^{6}$	−8.10	29.22	919.0	$1.93\times 10^{10}$	101.48	97.71

**Table 2:** List of attributes and how they were obtained.

Input/Output Attribute	Range	Source of Information
Metal	Au or Ag	Manufacturer
Size (radius, nm)	5–10	Manufacturer
Size error (nm)	1–2	Manufacturer
DNA length (Å)	126.52–330.52	[20,54,72]
Orientation angle (°)	0–39	[20,72]
Orientation error (°)	3–4	[20,72]
Theoretical length (Å)	98.32451–330.52	Supplementary Section S4
Length error (Å)	2.41–10.88603	Supplementary Section S4
Observed fluorescence	0.21325–1.57701	Experimental data
Theoretical NSET quench	2.79 × 10^−4^–0.9706	Calculated
Theoretical FRET quench	0.0028–0.99796	Calculated
α	2/3	[28]
*C*	0–2	[35,66,67]
$E_{2,1}$	−11.50–−4.75	Calculated
$e_{2,2}$	0.97–3.80	Calculated
$e_{2}^{2}$	34.11–133.23	Calculated
Overlap integral	1.37 × 10^18^–6.56 × 10^19^	Calculated
NSET $D_{0}$	36.57–234.89	Calculated
FRET $D_{0}$	98.43–275.89	Calculated
Fluorescence quenching	−0.57701–0.78675	Experimental data

**Table 3:** Optimized variables and error results.

EGS #	Ranges	*C*	α	MRE (Min)	RMSE (Min)
1	0.0≤α≤4.0 and 0.1≤C≤2.0	2	0.1	58.87231731	0.316617232
2	0.04≤α≤2.0 and 0.1≤C≤2.0	2	0.072943	40.74838502	0.247894406
3	0.04≤α≤0.1 and 0.1≤C≤2.0	2	0.0736	40.6450357	0.24876574

**Table 4:** Correlation coefficient ($R^{2}$) values for 2/3rd training and 1/3rd testing of the machine learning algorithms after standardization.

	Run Train	Run Test	5 CV Train	5 CV Test	10% Train ^1^	10% Test	10% Valid
MLP	0.99946	0.99749	0.99900	0.98764	0.99892	0.98678	0.99903
Lasso	0.99988	0.99986	0.99970	0.99972	0.99987	0.99930	0.99999

^1^ Here, 10% of the input and output attributes were randomly taken out for a validation parameter before calculation.

## Data Availability

The input and output data will be made available upon request. This includes experimental fluorescence quenching data.

## Supplementary Materials

The following supporting information can be downloaded at: https://www.mdpi.com/article/10.3390/nano14211741/s1, Figure S1: Gold dielectric components comparison; Figure S2: Silver dielectric components comparison; Figure S3: Copper dielectric components comparison; Figure S4: Pictoral diagram of oligonucleotide component lengths; Figure S5: Processing of emission spectrums; Figure S6: Silver nanoparticle aggregation example; Table S1: DNA Sequences; Table S2: Metal’s dielectric equation values. (References [74,75,76,77,78,79,80,81,82,83,84] are cited in Supplementary Materials).

## Abbreviations

The following abbreviations are used in this manuscript:

NSET	Nanometal surface energy transfer
FRET	Förster resonance energy transfer
AI/ML	Artificial intelligence/machine learning
DNA	Deoxyribonucleic acid
TET	Tetrachlorofulorescein
TCEP	Tris(2-carboxyethyl)phosphine
AuNP	Gold nanoparticle
AgNP	Silver nanoparticle
PB	Phosphate buffer
PBS	Phosphate buffer saline
bp	Base pair (DNA)
PEG	Polyethylene glycol
NP	Nanoparticle
PRET	Plasmon resonant energy transfer
MRE	Mean relative error
RMSE	Root mean squared error
EGS	Exhaustive grid search
MLP	Multilayer perceptron
CV	Cross-validation
Lasso	Least absolute shrinkage and selection operator
