# Supplementary material for: Optimization and Multimachine Learning Algorithms to Predict Nanometal Surface Area Transfer Parameters for Gold and Silver Nanoparticles
Source: Nanomaterials (Basel). 2024 Oct 30;14(21):1741. doi: 10.3390/nano14211741 (PMC11547468; doi:10.3390/nano14211741)
Supplement: Supplementary file 1 [file nanomaterials-14-01741-s001.zip › nanomaterials-3241163-supplementary.pdf]

Supplemental Material

1. DNA Strands

Name	Sequence (5'-3')	Extinction Coefficient (L*mol-1*cm-1)
AuThiol-20mer	/5ThioMC6-D//iSp9/ATCACTAGTGAATTCGCGGC	193000
TETDye-20mer	/5TET//iSp9/G CCG CGA ATT CAC TAG TGA T	208700
Atto425Dye-20mer	/5ATTO425N//iSp9/G CCG CGA ATT CAC TAG TGA T	200950
AuThiol-40mer	/5ThioMC6-D//iSp9/A TCA CTA GTG AAT TCG CGG CCG CCT GCA GGT CGA CCA TAT	378400
TETDye-40mer	/5TET//iSp9/A TAT GGT CGA CCT GCA GGC GGC CGC GAA TTC ACT AGT GAT	400300
Atto425Dye-40mer	/5ATTO425N//iSp9/A TAT GGT CGA CCT GCA GGC GGC CGC GAA TTC ACT AGT GAT	392550
AuThiol-60mer	/5ThioMC6-D//iSp9/A AGG AAT AGA GAC AAA GCC ACT CAT ATG AGT GGA ATA GAG ACA AAG CCA CTC ATA TGA GT	629900
TETDye-60mer	/5TET//iSp9/A CTC ATA TGA GTG GCT TTG TCT CTA TTC CAC TCA TAT GAG TGG CTT TGT CTC TAT TCC TT	564000
Atto425Dye-60mer	/5ATTO425N//iSp9/A CTC ATA TGA GTG GCT TTG TCT CTA TTC CAC TCA TAT GAG TGG CTT TGT CTC TAT TCC TT	556250
AuThiol-80mer	/5ThioMC6-D//iSp9/A TCA CTA GTG AAT TCG CGG CCG CCT GCA GGT CGA CCA TAT ATC ACT AGT GAA TTC GCG GCC GCC TGC AGG TCG ACC ATA T	756100
TETDye-80mer	/5TET//iSp9/A TAT GGT CGA CCT GCA GGC GGC CGC GAA TTC ACT AGT GAT ATA TGG TCG ACC TGC AGG CGG CCG CGA ATT CAC TAG TGA T	783600
Atto425Dye-80mer	/5ATTO425N//iSp9/A TAT GGT CGA CCT GCA GGC GGC CGC GAA TTC ACT AGT GAT ATA TGG TCG ACC TGC AGG CGG CCG CGA ATT CAC TAG TGA T	775850

2. Dielectric Function Models

Described in this section are the models considered in the computation section for the NSET calculation.

Drude: Standard equation used to determine the dielectric functions of a metal nanoparticle.[2]

$$\varepsilon_{Drude} = \varepsilon_{\infty} - \frac{{\omega_p}^2}{\omega^2 + {\Gamma_r}^2} + i\frac{\Gamma_r {\omega_p}^2}{\omega(\omega^2 + {\Gamma_r}^2)}$$

Breshike *et al*: To account for the interband contributions of gold metal, the following term was added to the standard Drude terms for determining the dielectric functions of the metal nanoparticles. A_i, ω_i, Φ_i, and Γ_i are fit parameters in frequency given by the authors.[3]

$$\varepsilon_{IB} = \sum_{i=1,2} \frac{A_i}{\omega_i}\left[\frac{e^{i\Phi_i}}{{\omega_i}^{-1} - \omega^{-1} - \Gamma_i^{-1}} + \frac{e^{-i\Phi_i}}{{\omega_i}^{-1} + \omega^{-1} + \Gamma_i^{-1}}\right]$$

Derkachova *et al*: Uses a logistic function to append to the Drude imaginary term ($\varepsilon_2(\omega) = \varepsilon_{Drude}(\omega) + i\Delta\varepsilon^{(Au)}(\omega)$) where A = 5.6 eV and Δ = 0.17 eV are the fit parameters to the Johnson & Cristy experimental data set.[4, 5]

$$\Delta\varepsilon^{(Au)}(\omega) = \frac{A}{1 + \exp\left(-(\omega - \omega_c)/\Delta\right)}$$

Size-Modified Drude: A ratio of the real and imaginary size-dependent Drude equation terms over those same Drude terms at infinite size (r = ∞, bulk). The obtained ratio is then applied as a multiplication factor to the Drude function for the metals at all wavelengths.

$$\varepsilon_{i,Multiplier} = \frac{\varepsilon_{i,radius}}{\varepsilon_{i,\infty}} = \frac{\varepsilon_{\infty} - \dfrac{{\omega_p}^2}{\omega^2 + {\Gamma_r}^2}}{\varepsilon_{\infty} - \dfrac{{\omega_p}^2}{\omega^2 + {\Gamma_{\infty}}^2}}, \frac{i\dfrac{\Gamma_r {\omega_p}^2}{\omega\left(\omega^2 + {\Gamma_r}^2\right)}}{i\dfrac{\Gamma_{\infty} {\omega_p}^2}{\omega\left(\omega^2 + {\Gamma_{\infty}}^2\right)}}$$

$$\varepsilon_{Mod-Drude} = \varepsilon_{Drude} * \varepsilon_{i,Multiplier}$$

Modified Bulk Dielectric: A ratio of the real and imaginary size-dependent Drude equation terms over those same Drude terms at infinite size (r = ∞, bulk). The obtained ratio is then applied to the experimentally found dielectric functions of the metals smoothed with a spline fit found by Johnson & Christy.[4]

$$\varepsilon_{Mod-Dieletric} = \varepsilon_{Dieletric} * \varepsilon_{i,Multiplier}$$

3. Dielectric Function Computation Comparison

With the NSET equation, a proper representation of the dielectric function for the metallic nanoparticles is critical for the formulation of a correct Förster distance, d_0. While thin films of the metals have been used to find the bulk optical properties of the material, special consideration needs to be made to account for the size effects of metallic nanoparticles. Presented here are bulk dielectric functions of gold compared to 5 different models for the metal dielectric functions: a pure Drude model, the Derkachova model, the Breshike model, and size-modified Drude and size-modified bulk dielectric functions.[3, 5]

Using gold as a foundation for the observations of other metal dielectric functions, a few experiments have found the optical properties of single nanoparticles.[6, 7] Using these references as a benchmark, we can then observe if a similar trend occurs for the different optical model presented in these findings. Below are the dielectric functions of gold nanoparticles of different sizes.

As stated in numerous other findings, interband transitions make significant contributions in the 300-500 nm wavelength region and cause the discrepancy from the Drude model in this region for the gold dielectric.[4, 8] To account for this difference, Derkachova *et al*[5] propose a logistic function to append to the Drude imaginary term ($\varepsilon_2(\omega) = \varepsilon_{Drude}(\omega) + i\Delta\varepsilon^{(Au)}(\omega)$) applied to the Johnson & Cristy experimental data set.[4] This logistic function is accurate for the region of 1-3 eV, which allows for accurate representation of the dielectric function at the emission wavelength of the TET dye at 539 nm (2.3 eV). The other necessary terms for the gold dielectric are taken from Oubre and Nordlander,[9] with ε_0 = 9.5 eV, Γ_∞ = 0.06909 eV, and ω_p = 8.9488 eV.

Once the dielectric components were determined, the extinction and absorption spectra for the gold nanoparticles were determined. The Bessel and Hankel functions were calculated to 15th order from the Mie Scattering equation.[10] The absorbance spectrum was then calculated and modified to fit the experimentally found relation described by Liu et al.[11] The wavelengths for the emission of the donor molecules were converted to eVs in the Python calculations to keep units consistent for the computation.

The main absorbance peak for gold nanoparticles is found in the region between 500-600 nm. Looking at this region, the Derkachova model follows more closely with bulk gold dielectric functions than the Drude or Breshike models across the different nanoparticle sizes. The Drude and modified Drude models do not follow the bulk gold until a wavelength of nearly 600 nm. The modified dielectric function exhibits the same characteristics of the Derkachova model while retaining the bulk gold measurement properties.

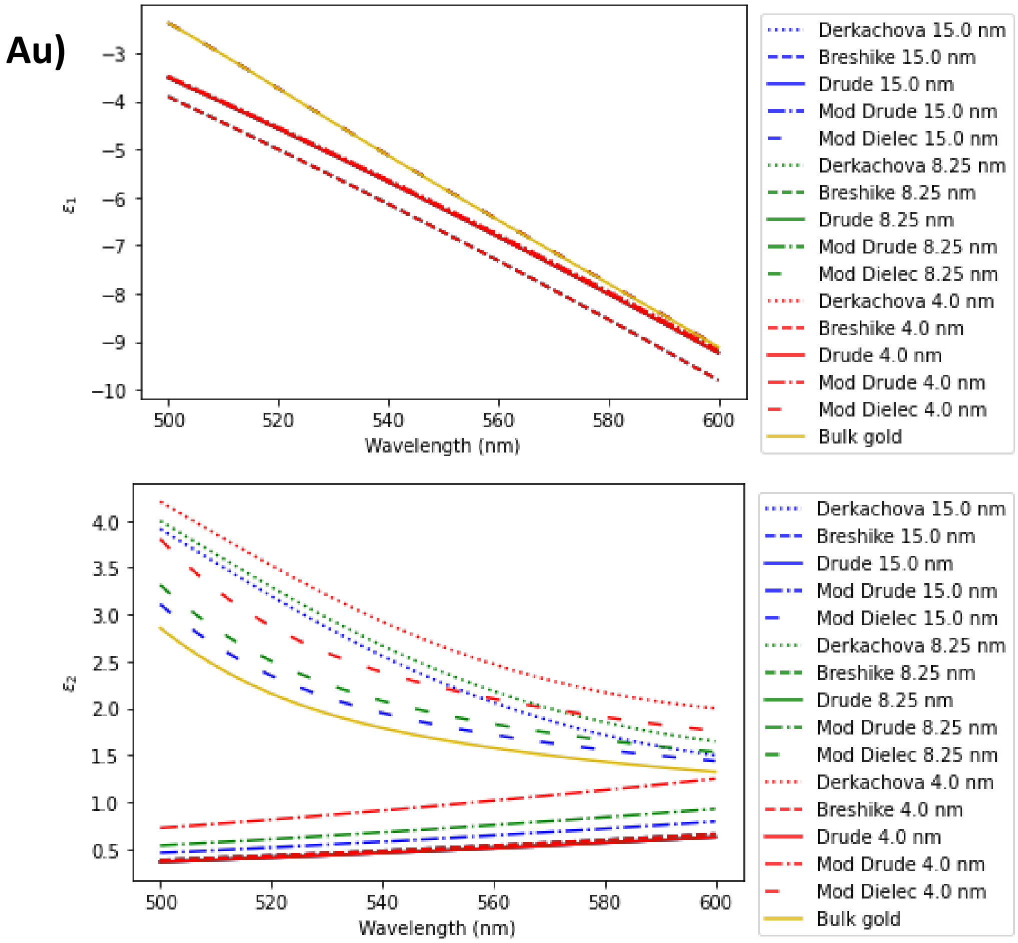

Figure S1: Top) Real component of gold's dielectric function from experimental (Bulk, Johnson & Christy) compared to the different size-dependent models. Bottom) Imaginary component of the dielectric function of those same data points.

For gold, the Derkachova[5] and the modified experimental dielectric function values most closely follow the values presented experimentally for gold nanoparticles.[6] However, the Drude model is based on the assumption that each individual metal atom has a single valency state.[2] Multivalent atoms require quantum mechanical computations to preciously model the dielectric functions. However, first approximating the Drude model is still generally used for metals considered for this work.

Beyond gold, other metals do not have the same attention to the mapping of the dielectric functions. As such, the modified experimental dielectric function from Equation 8 is used where the same trends in the dielectric function of gold are mirrored to nanoparticles of the different metals. These dielectric function graphs are displayed here for completeness.[3, 12-14] A table of the relevant dielectric values is also given in Table S*1* for all of the metals initially considered for this work.

Ag)

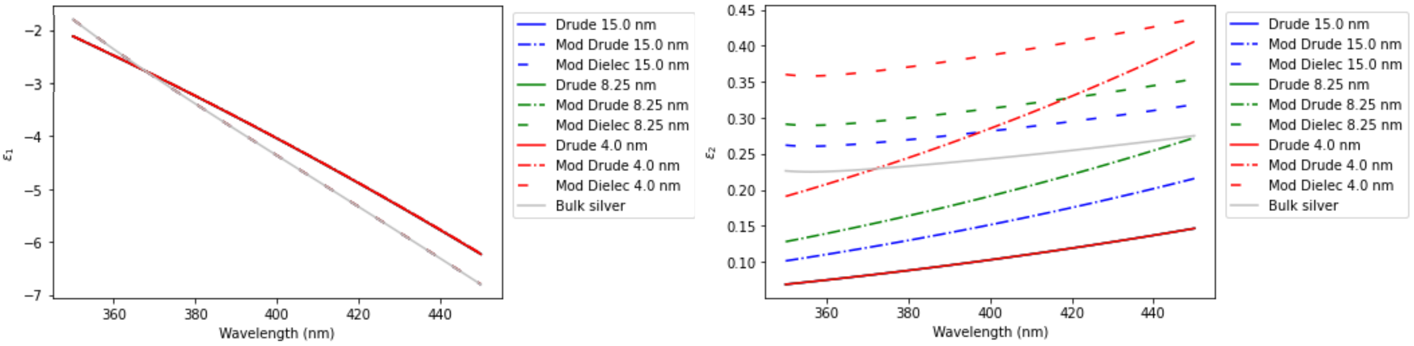

Figure S2: Real (left) and imaginary (right) dielectric components of silver between the experimental data (bulk, Johnson & Christy) and the different computed models.

Cu)

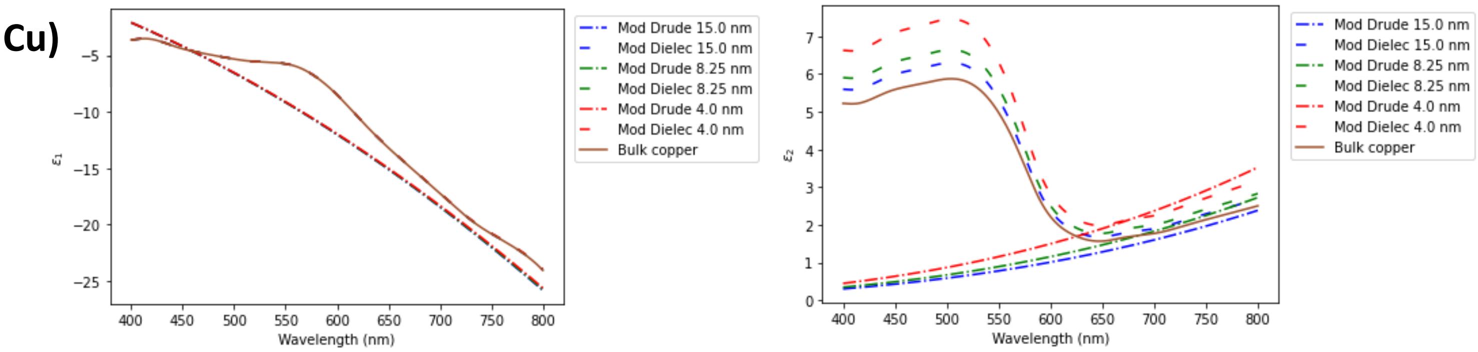

Figure S3: Real (left) and imaginary (right) dielectric components of copper between the experimental data (bulk, Palik[1]) and the different computed models.

Table S1: Values used in dielectric equations for the different metals. ε_∞ values are from fitting of the Drude model to the dielectric function if no reference is given.

	ε_∞ (eV)	ω_p (eV)	Γ_∞ (eV)	v_{fermi} (10^{15}nm/s)
Gold	9.5[9]	8.95[9]	0.0691[9]	1.40[2]
Silver	4.2	8.90[15]	0.0387[15]	1.39[2]
Platinum	1.2	5.15[14]	0.0692[16]	0.90[17]
Copper	5.9	8.76[13]	0.0955[13]	1.57[2]
Nickle	-	15.92[14]	0.0436[16]	0.23[18]
Palladium	-	5.45[14]	0.0154[16]	1.54[19]
Aluminum	-	12.04[13]	0.1287[13]	2.03[2]
Tungsten	-	13.22[20]	0.0640[16]	0.97[18]

4. DNA Length Calculation and TET Distance Error Calculations

The lengths of the DNA strands were calculated using the Clegg model for the base pair length, where the length of each base pair was estimated to be ~3.4 Å.[21] Assuming a ridged structure for the double stranded DNA duplex, the added length for the spacers (iSp9), thiol attachment (5ThiolM6-D), and the TET/Atto425 dye molecule.

For gold nanoparticles, the self-assembled DNA molecules can tilt at a metal surface or even electrostatically interact with the surface. This tilt effect may cause a discrepancy in the donor-acceptor distance for calculations of the energy transfer values. Using the packing density and tilt angle is gathered from Li for the different sized gold nanoparticles.[3, 22, 23] The calculated distances between the donor-acceptor pairs is thus calculated, along with the calculations for the maximum and minimum possible distances from the given errors for the different components.

Silver and platinum nanoparticles were assumed to have a similar packing and degree of tilt for the same sized nanoparticles, though a study of the packing for these materials would be beneficial for future work.

DNA Length	DNA Length (Å)	10 nm AuNP (θ=0±2)	NP Size Error (nm)	Error Max	Error Min
20 bp	126.52	126.52	1	126.52	126.44293
40 bp	194.52	194.52	1	194.52	194.4015
60 bp	262.52	262.52	1	262.52	262.36008
80 bp	330.52	330.52	1	330.52	330.31866

DNA Length	DNA Length (Å)	20 nm AuNP (θ=39±3)	NP Size Error (nm)	Error Max	Error Min
20 bp	126.52	98.32451	1	102.35683	94.02268
40 bp	194.52	151.17043	1	157.36999	144.55653
60 bp	262.52	204.01636	1	212.38314	195.09038
80 bp	330.52	256.86228	1	267.3963	245.62423

DNA Length	DNA Length (Å)	40 nm AuNP (θ=53±10)	NP Size Error (nm)	Error Max	Error Min
20 bp	126.52	76.14164	1.5	92.53087	57.43888
40 bp	194.52	117.06506	1.5	142.26292	88.31023
60 bp	262.52	157.98848	1.5	191.99497	119.18159
80 bp	330.52	198.9119	1.5	241.72703	150.05294

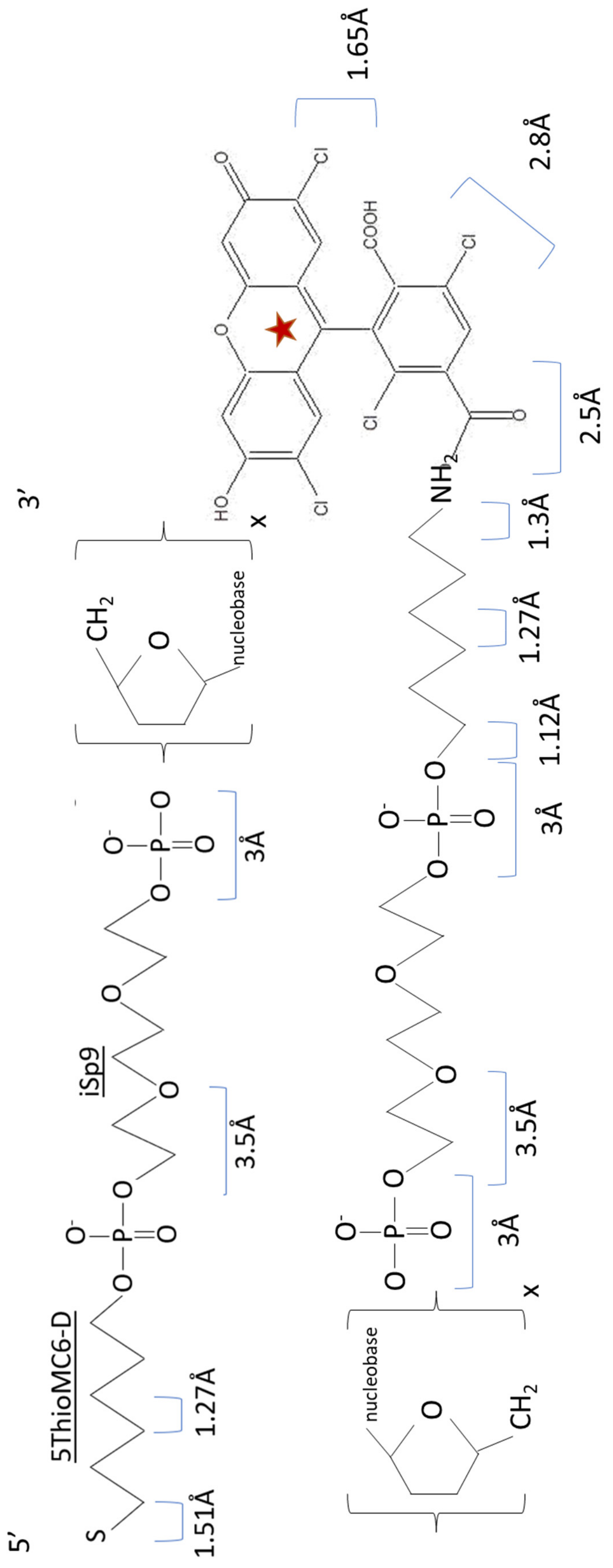

Figure S4: Pictorial Diagram of the lengths between the different oligonucleotide components.

Component Lengths:

- 5ThiolMC6-D ≈ 12.1 Å
- iSp9 ≈ 14.5 Å
- Base pair ≈ 3.4 Å/per
- TET centroid ≈ 17.42 Å

Equation for Distances:

- 58.52 + 3.4(# of bp) Å

Estimated D-A Distances:

- 20mer, ds = 126.52 Å
- 40mer, ds = 194.52 Å
- 60mer, ds = 262.52 Å
- 80mer, ds = 330.52 Å

5. Emission spectrum processing

To account for scattering and other effects, baseline absorbance and emission measurements were taken for the varying sized nanoparticles. Unmodified nanoparticles at roughly the same concentration as the NP-DNA-dye solutions were examined. From the absorbance measurements, the concentration of the unmodified nanoparticle solution was calculated at $\lambda = 450$ nm using the extinction values found by Haiss et al.[24] The DNA-conjugated nanoparticles underwent an identical concentration determination since the dsDNA-TET strands had minimal absorbance of 450 nm. A ratio was found between the two concentrations and then the scaling value was applied to the emission spectrum of the unmodified nanoparticles. The scaled background emission spectrum was then subtracted from the NP-DNA-TET emission spectrum to determine the emission of the TET dye alone. Thus, the effects of scattering and instrumental noise would be diminished in the resulting emission count used for determining the quenching efficiency of the NP-dye system.

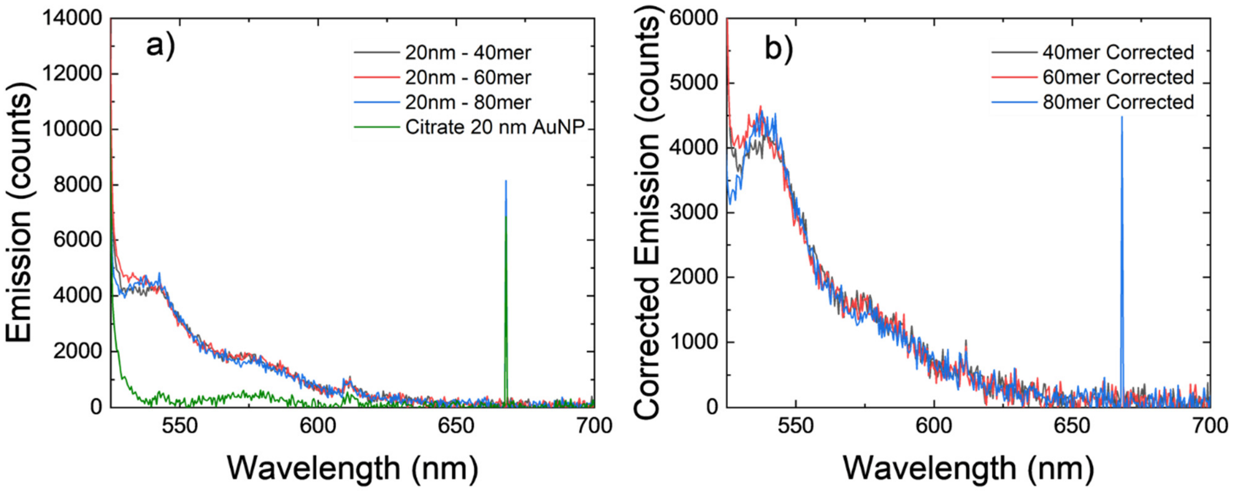

Figure S5: a) Emission spectrum of DNA-conjugated 20 nm gold nanoparticle solutions compared to the emission spectrum of citrate-coated 20 nm gold nanoparticles. Once the concentrations of the DNA-conjugated 20 nm gold nanoparticle solutions were determined, b) the original emission measurements had the modified baseline emission spectrum subtracted at each wavelength to get the corrected spectrum. The corrected spectra were then used to determine the quenching observed by the AuNP-TET pairings.

For the 10 nm nanoparticles, there was almost no detectable scattering from the emission measurements. The 20 nm nanoparticles had some significant scattering that can be seen in Figure S5. The scattering for the 40 nm gold nanoparticles drowns out the signal from the TET emissions. From what was learned with the gold nanoparticles, only 10 nm silver nanoparticles were attempted for the NSET measurements.

6. Silver Nanoparticle Trials

For the 10 nm silver nanoparticles, some aggregation was seen during the conjugation process as well as with the addition of the conjugate DNA-TET strands, as seen by the red shifting of the main plasmon peak in Figure 3. Aggregated silver nanoparticle clusters act in a manner like a larger sized silver nanoparticle, exhibiting a red shifting of the main plasmon peak seen in silver nanoparticles (Figure S6). The greater overlap and extinction of the larger silver clusters would increase the NSET and FRET exhibited by those nanoparticles and cause quenching beyond what is expected.

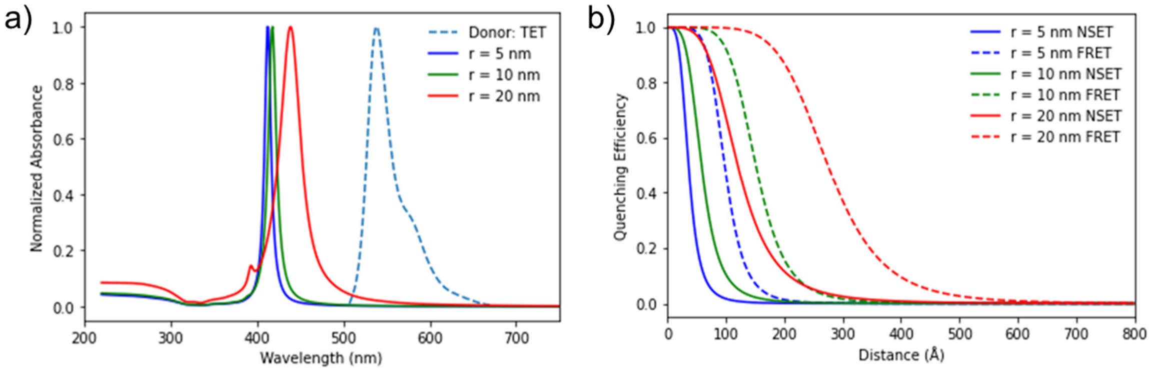

Figure S6: Effects of aggregation on silver nanoparticles. Effectively larger silver clusters would act in a manner like a larger silver nanoparticle. A) The larger nanoparticle would have a plasmon peak red shifted, causing a larger overlap with the TET dye used in experiments. B) The larger clusters would have an increased quenching on the TET emissions as well.

References

1. Palik, E. D., *Handbook of Optical Constants of Solids*. Academic Press: 1985; Vol. 1.
2. Ashcroft, N. W.; Mermin, N. D., *Solid State Physics*. Holt, Rinehart and Winston: 1976.
3. Breshike, C. J.; Riskowski, R. A.; Strouse, G. F., Leaving Förster Resonance Energy Transfer Behind: Nanometal Surface Energy Transfer Predicts the Size-Enhanced Energy Coupling between a Metal Nanoparticle and an Emitting Dipole. *The Journal of Physical Chemistry C* **2013,** *117* (45), 23942-23949.
4. Johnson, P. B.; Christy, R. W., Optical Constants of the Noble Metals. *Physical Review B* **1972,** *6* (12), 4370-4379.
5. Derkachova, A.; Kolwas, K.; Demchenko, I., Dielectric Function for Gold in Plasmonics Applications: Size Dependence of Plasmon Resonance Frequencies and Damping Rates for Nanospheres. *Plasmonics* **2016,** *11*, 941-951.
6. Stoller, P.; Jacobsen, V.; Sandoghdar, V., Measurement of the complex dielectric constant of a single gold nanoparticle. *Opt. Lett.* **2006,** *31* (16), 2474-2476.
7. Descoteaux, M.; Sunnerberg, J. P.; Staii, C., Quantitative characterization of dielectric properties of nanoparticles using electrostatic force microscopy. *AIP Advances* **2020,** *10* (11), 115118.
8. Olmon, R. L.; Slovick, B.; Johnson, T. W.; Shelton, D.; Oh, S.-H.; Boreman, G. D.; Raschke, M. B., Optical dielectric function of gold. *Physical Review B* **2012,** *86* (23), 235147.

9. Oubre, C.; Nordlander, P., Optical Properties of Metallodielectric Nanostructures Calculated Using the Finite Difference Time Domain Method. *The Journal of Physical Chemistry B* **2004,** *108* (46), 17740-17747.
10. Demers, S. M. E.; Hsieh, L. J. H.; Shirazinejad, C. R.; Garcia, J. L. A.; Matthews, J. R.; Hafner, J. H., Ultraviolet Analysis of Gold Nanorod and Nanosphere Solutions. *The Journal of Physical Chemistry C* **2017,** *121* (9), 5201-5207.
11. Liu, X.; Atwater, M.; Wang, J.; Huo, Q., Extinction coefficient of gold nanoparticles with different sizes and different capping ligands. *Colloids and Surfaces B: Biointerfaces* **2007,** *58* (1), 3-7.
12. Yu, R.; Liz-Marzán, L.; Garcia de Abajo, J., Universal analytical modeling of plasmonic nanoparticles. *Chem. Soc. Rev.* **2017,** *46*.
13. Zeman, E. J.; Schatz, G. C., An accurate electromagnetic theory study of surface enhancement factors for silver, gold, copper, lithium, sodium, aluminum, gallium, indium, zinc, and cadmium. *The Journal of Physical Chemistry* **1987,** *91* (3), 634-643.
14. Ordal, M. A.; Bell, R. J.; Alexander, R. W.; Long, L. L.; Querry, M. R., Optical properties of fourteen metals in the infrared and far infrared: Al, Co, Cu, Au, Fe, Pb, Mo, Ni, Pd, Pt, Ag, Ti, V, and W. *Appl. Opt.* **1985,** *24* (24), 4493-4499.
15. Yang, H. U.; D'Archangel, J.; Sundheimer, M. L.; Tucker, E.; Boreman, G. D.; Raschke, M. B., Optical dielectric function of silver. *Physical Review B* **2015,** *91* (23), 235137.
16. Li, Y., *Plasmonic Optics: Theory and Applications*. SPIE Press: 2017.
17. Bordoloi, A.; Auluck, S., Electronic structure of platinum. *Journal of Physics F: Metal Physics* **2000,** *13*, 2101.
18. Gall, D., Electron mean free path in elemental metals. *Journal of Applied Physics* **2016,** *119* (8).
19. G. Crabtree, D. D., J. Ketterson, N. Sandesara, J. Vuillemin, FERMI RADII, FERMI VELOCITIES, AND MANY BODY ENHANCEMENT IN PALLADIUM. *Journal de Physique Colloques* **1978,** *39*, C6-1095-C6-1096.
20. Rakic, A. D.; Djurisic, A. B.; Elazar, J. M.; Majewski, M. L., Optical properties of metallic films for vertical-cavity optoelectronic devices. *Appl Opt* **1998,** *37* (22), 5271-83.
21. Clegg, R. M.; Murchie, A. I.; Zechel, A.; Lilley, D. M., Observing the helical geometry of double-stranded DNA in solution by fluorescence resonance energy transfer. *Proc Natl Acad Sci U S A* **1993,** *90* (7), 2994-8.
22. Hill, H. D.; Millstone, J. E.; Banholzer, M. J.; Mirkin, C. A., The role radius of curvature plays in thiolated oligonucleotide loading on gold nanoparticles. *ACS Nano* **2009,** *3* (2), 418-24.
23. Li, Z.; Niu, T.; Zhang, Z.; Chen, R.; Feng, G.; Bi, S., Exploration of the specific structural characteristics of thiol-modified single-stranded DNA self-assembled monolayers on gold by a simple model. *Biosensors and Bioelectronics* **2011,** *26* (11), 4564-4570.
24. Haiss, W.; Thanh, N. T. K.; Aveyard, J.; Fernig, D. G., Determination of Size and Concentration of Gold Nanoparticles from UV−Vis Spectra. *Analytical Chemistry* **2007,** *79* (11), 4215-4221.
